# p73 – NAV3 axis plays a critical role in suppression of colon cancer metastasis

**DOI:** 10.1038/s41389-020-0193-4

**Published:** 2020-02-06

**Authors:** Apoorva Uboveja, Yatendra Kumar Satija, Fouzia Siraj, Ira Sharma, Daman Saluja

**Affiliations:** 1 Dr. B.R. Ambedkar Centre for Biomedical Research, University of Delhi, New Delhi, 110007 India; 2 National Institute of Pathology (ICMR), Safdarjung Hospital Campus, New Delhi, 110029 India; 3 Present Address: CSIR-Indian Institute of Toxicology Research, Lucknow, 226001 India

**Keywords:** Extracellular matrix, Metastasis, DNA damage response

## Abstract

p73 is a member of the p53 tumor suppressor family, which transactivates p53-responsive genes and mediates DNA damage response. Recent evidences suggest that p73 exerts its tumor suppressor functions by suppressing metastasis, but the exact mechanism remains unknown. Here, we identify Navigator-3 (NAV3), a microtubule-binding protein, as a novel transcriptional target of p73, which gets upregulated by DNA damage in a p73-dependent manner and plays a vital role in p73-mediated inhibition of cancer cell invasion, migration, and metastasis. Induction of p73 in response to DNA damage leads to rapid increase in endogenous NAV3 mRNA and protein levels. Through bioinformatic analysis, we identified two p73-binding sites in NAV3 promoter. Consistent with this, p73 binding to NAV3 promoter was confirmed through luciferase, Chromatin Immunoprecipitation, and site-directed mutagenesis assays. Abrogation of NAV3 and p73 expression significantly increased the invasion and migration rate of colorectal cancer cells as confirmed by wound-healing, cell invasion, and cell migration assays. Also, knockdown of NAV3 decreased the expression of E-cadherin and increased the expression of other prominent mesenchymal markers such as N-cadherin, Snail, Vimentin, and Fibronectin. Immunohistochemistry analysis revealed the downregulation of both NAV3 and p73 expression in metastatic colon cancer tissues as compared to non-metastatic cancer tissues. Additionally, the expression pattern of NAV3 and p73 showed extensively significant correlation in both non-metastatic and metastatic human colon cancer tissue samples. Taken together, our study provide conclusive evidence that Navigator-3 is a direct transcriptional target of p73 and plays crucial role in response to genotoxic stress in p73-mediated inhibition of cancer cell invasion, migration, and metastasis.

## Introduction

The transcription factor p73 is a member of the p53 tumor suppressor family and shows substantial structural and functional homology with p53^1^. Analogous to p53, p73 is present at very low levels but it gets swiftly induced upon genotoxic stress^2^. p73 can bind to the p53 response elements and transactivate p53 target genes involved in cell cycle arrest and apoptotic cell death as well as mediate genotoxic stress response^2,3^. In addition, p73 is known to activate p53-independent target genes^4^ and p73 restoration elicits a p53-like tumor suppressive effect^5^. An extensive search of the p73 status in human primary tumors revealed that p73 mutations are detected in fewer than 0.5% of human cancers, whereas over 50% of cancers carry p53 mutations^6^, making p73 an attractive target for therapeutic intervention. Furthermore, unlike p53 gene, which shows only little alternative splicing, p73 gives rise to multiple isoforms, due to alternative promoter usage and differential mRNA splicing^7^. Functionally, the TAp73 isoforms closely mimic p53 in the ability to stimulate transcription of death genes and to trigger programmed cell death and has been shown to be a bona fide tumor suppressor whereas the DNp73 isoforms are strong inhibitors of transcriptionally active p73 and p53^8,9^.

p73 has been known to execute its tumor suppressive function by guarding the genomic stability and promoting cell cycle arrest, replicative senescence or apoptosis^10^. Data from several human tumors, including lung adenocarcinoma, transitional cell carcinoma of the bladder, osteosarcoma, mammary adenocarcinoma, and myelogenous leukemia, indicate that loss of p73 correlates with tumor formation^11,12^. It has been shown that TA-p73 null mice are prone to lung cancer, indicating that it functions as a tumor suppressor^13–15^. Moreover, a recent study revealed a 37% decrease in the levels of TAp73 mRNA in neoplastic tissue as compared to that of normal colon tissue, suggesting that p73 may play a role as a tumor suppressor in colorectal cancer (CRC) progression^16^. In addition to pro-arrest and pro-apoptotic roles, p73 also exerts its tumor suppressor function by suppressing metastasis^17^. Furthermore, p73 contributes to its anti-metastatic function by increasing the expression of Ago-1/2, which leads to an increase in the processing of miRNAs such as let-7, miR-134, miR-130b, miR-449a, miR-181d, and miR-379, which activate tumor suppressor mechanisms and suppress epithelial to mesenchymal transition (EMT), metastasis and cancer stem cell proliferation^18^. p73 was also shown to have inhibitory effects on the actin cytoskeleton dynamics and thereby cancer cell motility however, the exact mechanism remains to be explored^19^.

Neuron navigator 3 (NAV3) is one of the three members of the neuron navigator family, a human gene family which shares homology with unc-3, a cell guidance gene from *Caenorhabditis elegans*^20^. This gene family has been known to be involved in a variety of cellular processes, including protein degradation, signal transduction, regulation of gene expression, membrane fusion, microtubule dynamics, and cell migration^20^. Gene duplication during evolution may have led to formation of three human homologs located on chromosomes 1q32.1 (NAV1), 11p15.1 (NAV2), and 12q21.1 (NAV3)^20^. NAV3, in particular, is a microtubule binding protein usually downregulated in tumors and acts as a regulator of cell migration and invasion^21^. NAV3 protein contains various functional domains including a calponin homology (CH) domain, four coiled-coil (CC) domains, a cytoskeletal interacting domain (CSID), and an AAA-domain^20^. Succeeding induction by growth factors, NAV3 binds to the plus ends of microtubules and augments their polarized growth. Consistently, studies have shown that NAV3 attenuation trimmed microtubule growth, perpetuated growth factor signaling, prevented apoptosis, and elevated random cell migration^22^. Additionally, previous studies have identified NAV3 as a putative tumor suppressor in cutaneous T-cell lymphoma and in the associated lung tumors^21,23^. Low copy number of NAV3 in colon carcinomas correlated with poor prognosis or poor response to therapy^24^. Downregulation of NAV3 has been observed during the progression of many types of human tumors and is correlated with poor survival in patients with those cancers, suggesting that it has an anti-metastatic role^25^.

In the present study, we identified NAV3 as a direct transcriptional target of p73, which gets induced by genotoxic stress in a p73-dependent manner. Identification of p73 binding sites in NAV3 promoter region suggested that the expression of NAV3 is directly regulated by p73, which was further confirmed by chromatin immunoprecipitation (ChIP) and site-directed mutagenesis experiments. Additionally, depletion of NAV3 enhanced cell migration and invasion in a p73-dependent manner. Furthermore, Immunohistochemistry (IHC) analysis of non-metastatic and metastatic human colon cancer tissue samples revealed that NAV3 and p73 levels were significantly downregulated in metastatic tumor tissue samples as compared to non-metastatic tumor tissue samples. Taken together, these results provide evidence that p73 is necessary for NAV3 induction in response to genotoxic stress and that NAV3 plays a pivotal role in p73-mediated inhibition of cancer cell invasion, migration, and metastasis. Moreover, the p73-mediated regulation of NAV3 is completely independent of p53.

## Results

### Genotoxic stress results in upregulation of Navigator-3 (NAV3) in a p73-dependent manner

**Fig. 1:**
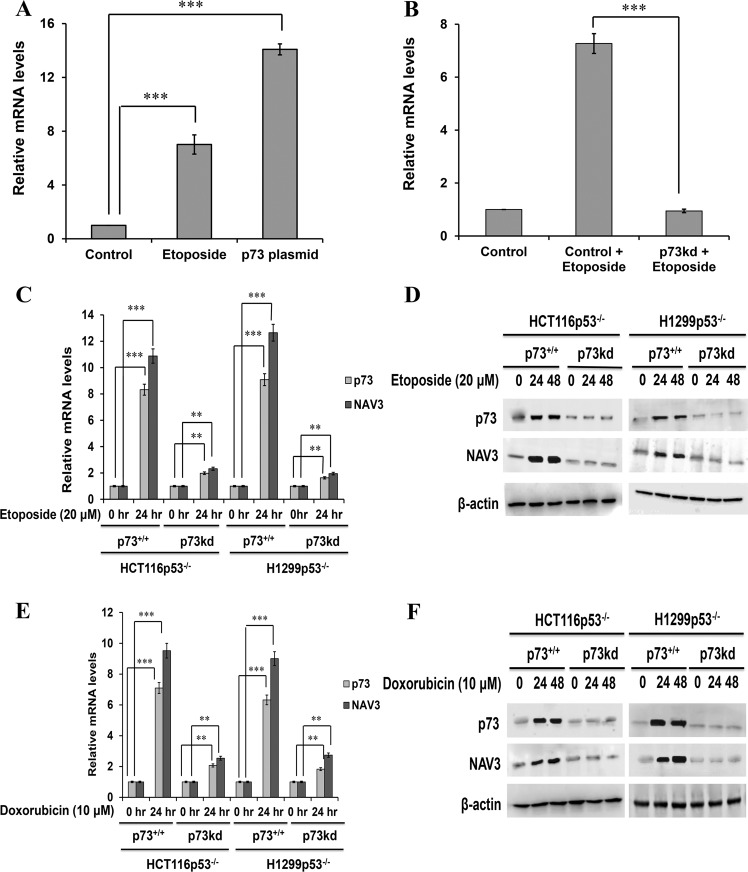
Genotoxic stress mediated upregulation of p73 and NAV3. **a**, **b** RT-qPCR for NAV3 in (**a**) untreated, etoposide (20 μM, 24 h) treated HCT116p53^−/−^ cells and p73 plasmid transfected HCT116p53^−/−^ cells and (**b**) etoposide (20 μM, 24 h) treated HCT116p53^−/−^p73^+/+^ (Control) and HCT116p53^−/−^p73kd (p73kd) cells, normalized with β-actin. The results of three independent experiments are presented as the mean ± SD (unpaired two-tailed Student’s t-test, ***P < 0.001). **c**, **d** RT-qPCR (**c**) for NAV3 and p73 and western (**d**) for NAV3, p73 and β-actin (loading control) in etoposide treated (20 μM) HCT116p53^−/−^p73^+/+^ (p73^+/+^), HCT116p53^−/−^p73kd (p73kd), H1299p53^−/−^p73^+/+^ (p73^+/+^) and H1299p53^−/−^p73kd (p73kd) cells for the indicated time points. The results (**c**) of three independent experiments are presented as the mean ± SD (unpaired two-tailed Student’s *t*-test, *P < 0.05, **P < 0.01, ***P < 0.001). **e**, **f** RT-qPCR (**e**) for NAV3 and p73 and western (**f**) for NAV3, p73 and β-actin (loading control) in doxorubicin treated (10 μM) HCT116p53^−/−^p73^+/+^ (p73^+/+^),HCT116p53^−/−^p73kd (p73kd), H1299p53^−/−^p73^+/+^ (p73^+/+^) and H1299p53^−/−^p73kd (p73kd) cells for the indicated time points. Fold changes were calculated after normalization with β-actin. The results (**e**) of three independent experiments are presented as the mean ± SD (unpaired two-tailed Student’s *t*-test, ***P < 0.001).

To ascertain the involvement of p73 in regulation of NAV3, a quantitative real-time PCR using etoposide (20 μM) as a mediator of genotoxic stress^26^ was performed in HCT116p53^−/−^ CRC cell line. Etoposide treatment resulted in upregulation of NAV3 mRNA levels as compared to unstressed cells (Fig. 1a). Furthermore, to overexpress p73, HCT116p53^−/−^ cells were transfected with a plasmid expressing p73, which also increased NAV3 mRNA levels as compared to that of control cells (Fig. 1a). Consistent with p73 expression, NAV3 protein levels were also significantly induced by etoposide treatment in HCT116p53^−/−^ cells (Supplementary Fig. S1A). To check whether p73 and NAV3 levels get induced by etoposide in other p53^−/−^ cell lines too, H1299p53^−/−^ cells were treated with etoposide (20 μM) for 24 and 48 h and western blot analysis was carried out. p73 and NAV3 were also found to be upregulated upon etoposide treatment in H1299p53^−/−^ cell lines upto 48 h as compared to that of control cells (Supplementary Fig. S1B). A stable p73 knockdown cell line (pooled puromycin-resistant population) was generated using a specific shRNA in HCT116p53^−/−^ cells and knockdown was confirmed by western blotting (Supplementary Fig. S1C). NAV3 mRNA levels were then checked in etoposide treated control and p73kd cells by quantitative real-time PCR and were found to be significantly decreased in p73kd cells as compared to control cells (Fig. 1b). Furthermore, we determined the kinetics of NAV3 expression upon etoposide treatment in Control and p73kd cells at various time points from 12 to 48 h. We found that enhanced expression of NAV3 mRNA was detected in control cells as early as 12 h and kept on increasing with increase in duration of genotoxic stress. However, p73kd abrogated the induction of NAV3 and thus, no increase was observed in NAV3 mRNA levels even after genotoxic stress (Supplementary Fig. S1D). Consistent with this observation, elevated NAV3 protein levels were observed after treatment with etoposide in a time-dependent manner in control cells. In contrast, no induction of NAV3 protein levels were observed in p73kd cells after treatment with etoposide for the indicated time-points (Supplementary Fig. S1E). Similarly, a stable p73 knockdown cell line (pooled puromycin-resistant population) was generated using a specific shRNA in H1299p53^−/−^ cells. NAV3 mRNA and protein levels were then checked in etoposide (20 μM) treated H1299p53^−/−^p73^+/+^ and H1299p53^−/−^p73kd cells by quantitative real-time PCR and western blotting respectively. NAV3 mRNA (Fig. 1c) and protein (Fig. 1d) levels were found to be significantly decreased in p73kd cells as compared to p73^+/+^ cells upon etoposide treatment upto 24 h. To further check whether p73 and NAV3 levels get induced by other genotoxic drugs, HCT116p53^−/−^p73^+/+^, HCT116p53^−/−^p73kd, H1299p53^−/−^p73^+/+^, and H1299p53^−/−^p73kd cells were treated with doxorubicin (10 μM) for 24 and 48 h. p73 and NAV3 mRNA (Fig. 1e) and protein (Fig. 1f) levels were found to be significantly decreased in p73kd cells as compared to p73^+/+^ cells in the cell lines. Taken together, these results indicate that p73 expression is required for increased expression of NAV3. To further examine whether p53 also regulates NAV3, p73 expression was abrogated by specific shRNA (pooled puromycin-resistant population) in HCT116p53^+/+^ cells (control cell line) and NAV3 mRNA levels in etoposide treated control and p73kd cells were quantified by quantitative real-time PCR. NAV3 mRNA levels were found to be significantly decreased in p73kd cells even in the presence of p53 (Supplementary Fig. S1F). Western blot analysis revealed that induction of NAV3 protein was abrogated after p73kd in HCT116p53^+/+^ cells as compared to that of control cells (Supplementary Fig. S1G), suggesting that p53 status does not affect NAV3 expression levels.

### Identification of putative p73 binding sites in the promoter region of Navigator-3 gene

Since our data indicate that NAV3 is a p73-responsive gene, and p73 primarily functions as a transcription factor that binds to target DNA sequences, we wanted to establish whether p73 binds to promoter of NAV3. We therefore scanned the human NAV3 genomic region spanning 2 kb upstream and 2 kb downstream of the Transcription Start Site (TSS) for potential p73-recognition sequences by using the TFBind software (tfbind.hgc.jp) and JASPAR database (jasper.genereg.net). Typically, one consensus p73 binding site consists of two 10 bp half sites- 5′- RRRC(A/T)(A/T)GYYY, where C(A/T)(A/T)G is the core sequence. The two half sites are separated by a spacer with upto 13 bases and the purine (R) and pyrimidine (Y) bases comprise the flanking sequence (Supplementary Fig. S2A). Eleven putative p73 half binding sites (BS) were identified in the upstream region (1949 bp) and nine sites were identified in the downstream region (2630 bp) with respect to the TSS (Supplementary Fig. S2B). In order to test the interaction of p73 with these putative half binding sites, the upstream region (1949 bp) was divided into two parts: U1 (−2269 to −1437; 833 bp) and U2 (−1456 to −341; 1116 bp) and the downstream region (2630 bp) was divided into two parts: D1 (+8 to +1101; 1094 bp) and D2 (+1958 to +2638; 681 bp) (Supplementary Fig. S2C).

### p73 directly binds to the NAV3 promoter

**Fig. 2:**
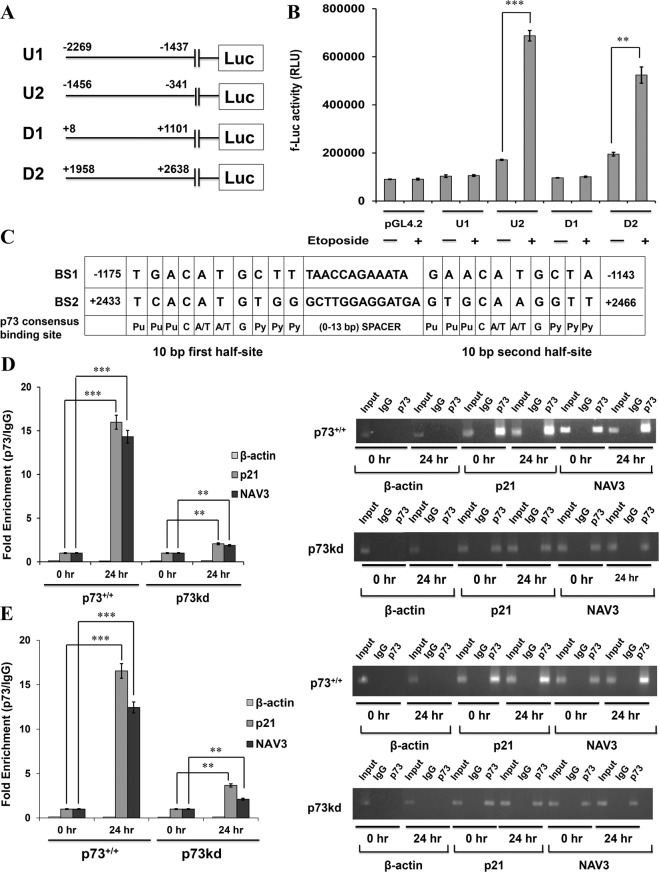
NAV3 promoter is a direct target of p73. **a** Schematic representation of NAV3 promoter regions cloned upstream of the luciferase reporter gene. **b** Luciferase assay in untreated and etoposide (20 μM, 48 h) treated HCT116p53^−/−^ (control) cells transfected with empty pGL4.2 luciferase vector, pGL4.2-NAV3 promoter-reporter constructs and pRLT-K loading control. The results are presented as mean ± SD of three independent experiments (unpaired two-tailed Student’s *t*-test, **P < 0.01, ***P < 0.001). **c** Schematic representation of two potential p73 binding sites, BS1 (−1175 to −1143) present in the U2 region and BS2 (+2433 to +2466) present in the D2 region of NAV3 promoter. **d**, **e** ChIP RT-qPCR performed in etoposide (20 μM) treated HCT116p53^−/−^p73^+/+^ (control) and HCT116p53^−/−^p73kd (p73kd) cells for the indicated time points with antibody against p73 and primers specific to BSS1 region (−1257 to −1042) containing BS1 binding site present in U2 region (**d**) and BSS2 region (+2396 to +2590) containing BS2 binding site present in D2 region (**e**) of NAV3 promoter. β-actin was used as a negative control and p21 as a positive control. The results are presented as mean ± SD of three independent experiments (paired two-tailed Student’s *t*-test, *P < 0.05, **P < 0.01, ***P < 0.001).

To further pinpoint the functional p73 binding site present in NAV3 promoter, four fragments that contain these potential p73-binding sites—U1, U2, D1, and D2 were cloned upstream of a luciferase reporter gene into pGL4.2 firefly luciferase reporter vector. These different constructs were transfected into HCT116p53^−/−^ cells, along with an internal loading control vector (pRL-TK) that expresses Renilla luciferase (Fig. 2a). Luciferase activity was measured post 24 h etoposide treatment. The empty luciferase vector was used as negative control. Reporter constructs containing the U2 region (−1456 to −341) and the D2 (+1958 to +2638) region exhibited significantly greater luciferase activity upon genotoxic stress when compared to control (Fig. 2b). On the other hand, constructs containing U1 and D1 region showed no change in luciferase activity upon etoposide treatment as compared to control (Fig. 2b). Similarly, luciferase activity of the different reporter constructs was also checked in H1299p53^−/−^ cells and reporter constructs containing the U2 and D2 region exhibited significantly higher luciferase activity upon genotoxic stress as compared to the reporter constructs containing U1 and D1 regions of NAV3 promoter (Supplementary Fig. S3A). We found two complete p73 binding sites BS1 (−1175 to −1143) and BS2 (+2433 to +2466) located in the U2 and D2 regions respectively (Fig. 2c), having minimum mismatches with the p73 consensus binding sequence.

To confirm the direct interaction of p73 with NAV3 promoter in vivo and to identify the binding site, ChIP assays were performed. HCT116p53^−/−^p73^+/+^ and p73kd cells were treated with etoposide for various time points and ChIP was then performed using p73 antibody. RT-qPCR was then performed on the immunoprecipitated DNA and on total input DNA using primers that flanked the putative p73 binding site BS1; BSS1 (−1257 to −1042) present in the U2 region of the NAV3 promoter, to produce an expected 215-bp product and BS2 site; BSS2 (+2396 to +2590) present in the D2 region of the NAV3 promoter, to produce an expected 194-bp product. β-actin was used as a negative control and p21 as a positive control. Endogenous p73 was found to bind to BS1 (Fig. 2d, Supplementary Fig. S3B) as well as to BS2 (Fig. 2e, Supplementary Fig. S3C) and the fold enrichment increased with duration of etoposide treatment. As expected, no significant increase in binding to NAV3 promoter (BS1 and BS2) was observed in p73kd cells treated with etoposide. This strongly suggested that p73 directly binds the predicted sequences (BS1 and BS2) in the NAV3 promoter to activate transcription of NAV3 in vivo.

### Site-directed mutagenesis confirms p73 binding sites in NAV3 promoter

**Fig. 3:**
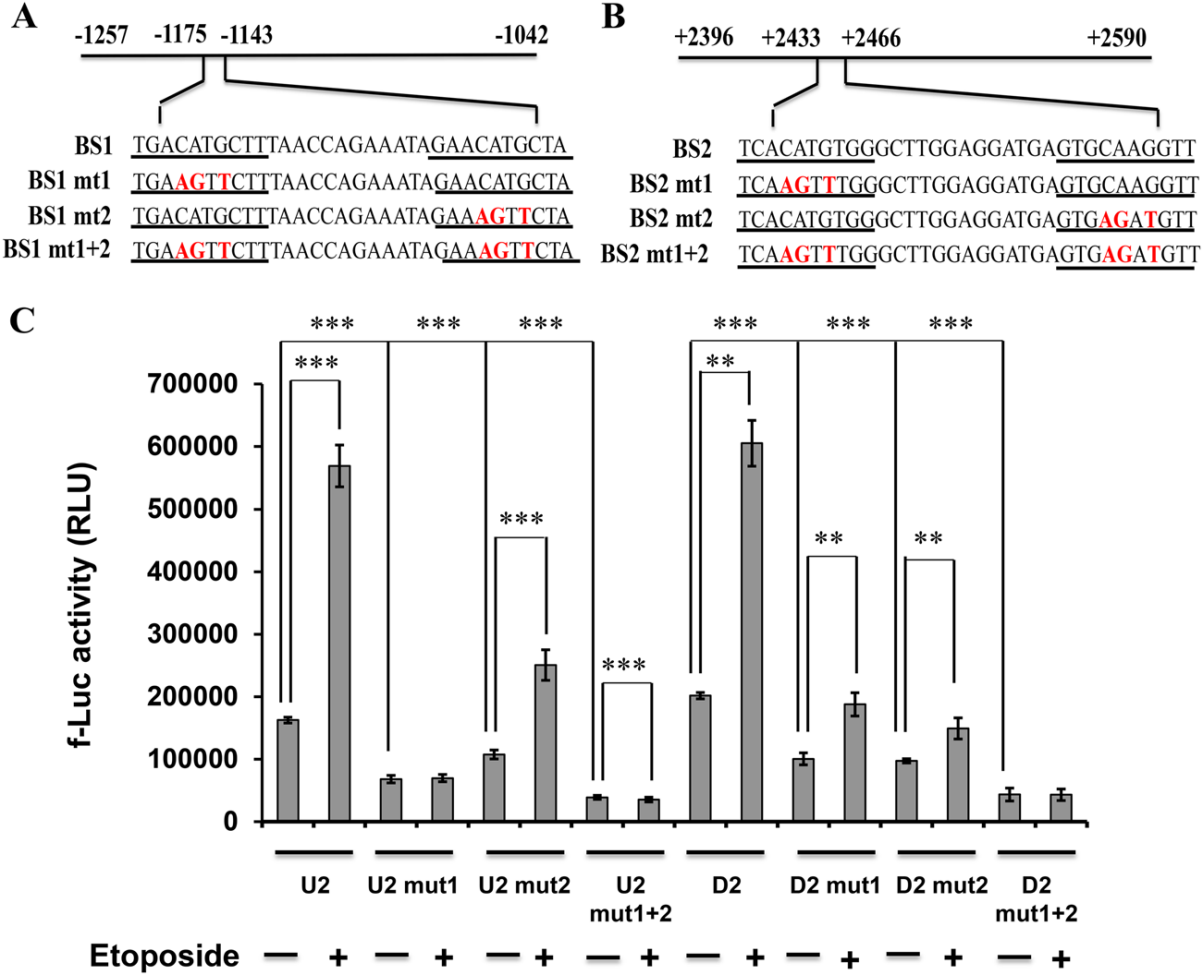
Site-directed mutagenesis reveals the correct binding site of p73. **a**, **b** Schematic representation of luciferase reporter plasmids with mutations (boldface) in BS1 in U2 region (**a**) and BS2 in D2 region (**b**) of p73 binding site (underlined) in NAV3 promoter. **c** Luciferase assay in untreated (control) and etoposide treated (20 μM) HCT116p53^−/−^p73^+/+^ cells transfected with plasmids carrying mutations in BS1 and BS2 p73 binding sites. The results are presented as mean ± SD of three independent experiments (unpaired two-tailed Student’s *t*-test, **P < 0.01, ***P < 0.001).

To further explore the role of the predicted p73 binding sites BS1 and BS2 in mediating p73 induced NAV3 promoter activity, luciferase reporter constructs were generated with mutations created by nucleotide substitutions within the core sequence of BS1 (Fig. 3a) and BS2 (Fig. 3b) p73 binding sites. Luciferase assay was performed 24 h post etoposide treatment. Mutation of CATG to AGTT in the first half-site of BS1 binding site (U2 mut1) led to a 2.3 fold reduction in the luciferase activity and a similar mutation in the second half-site of BS1 binding site (U2 mut2) resulted in a 1.5 fold reduction even in the absence of etoposide treatment. A 5.65 fold reduction in the luciferase activity of U2 mut1 was observed after treatment with etoposide whereas a 3.56 fold reduction was observed in case of U2 mut2 after treatment with etoposide, suggesting that the first half-site of BS1 is more potent for p73 binding than the second half site of BS1. Mutation of both the half sites (U2 mut1 + 2) abrogated almost completely the effect of p73 as negligible luciferase activity was seen (Fig. 3c). Similarly, in the absence of etoposide treatment, mutation of CATG to AGTT in the first half-site of BS2 binding site (D2 mut1) led to a 2.01 fold decline in the luciferase activity. Mutation of CAAG to AGAT in the second half-site of BS2 binding site (D2 mut2) led to a similar reduction (2.07 fold) in luciferase activity. Even in the etoposide treated cells, mutations in the two half-sites of BS2 gave similar effect with 3.0 fold and 3.5 fold reduction in luciferase activity with D2 mut1 and D2 mut2 respectively. Mutations in both the half-sites (D2 mut1 + 2) abolished almost completely p73 responsiveness (Fig. 3c), suggesting that both the half-sites of BS2 equally contribute towards p73 binding. These results confirm that p73 interacts specifically with both BS1 and BS2 binding sites present in the NAV3 promoter to mediate its expression.

### Knockdown of NAV3 increases the migration and invasion capacity of CRC cells

**Fig. 4:**
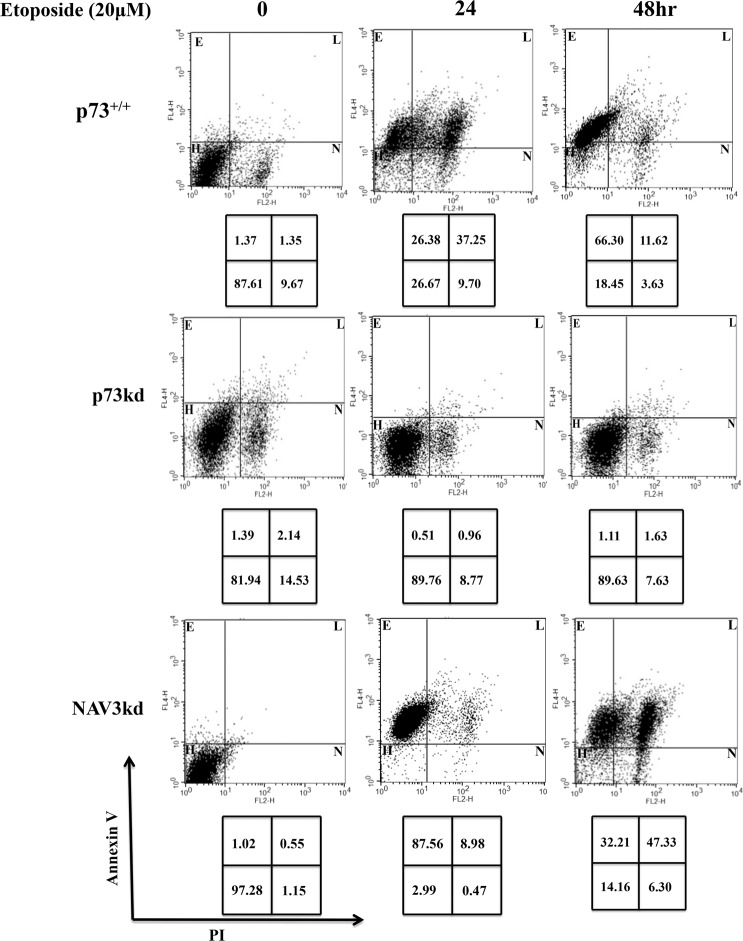
Role of NAV3 in p73-mediated apoptosis. HCT116p53^−/−^p73^+/+^ (p73^+/+^), HCT116p53^−/−^p73kd (p73kd) and HCT116p53^−/−^p73^+/+^NAV3kd (NAV3kd) cells were treated with etoposide (20 μM) for 24 and 48 h, followed by Annexin V-PI analysis of apoptosis by flow cytometer is presented. The experiment was repeated three times.

**Fig. 5:**
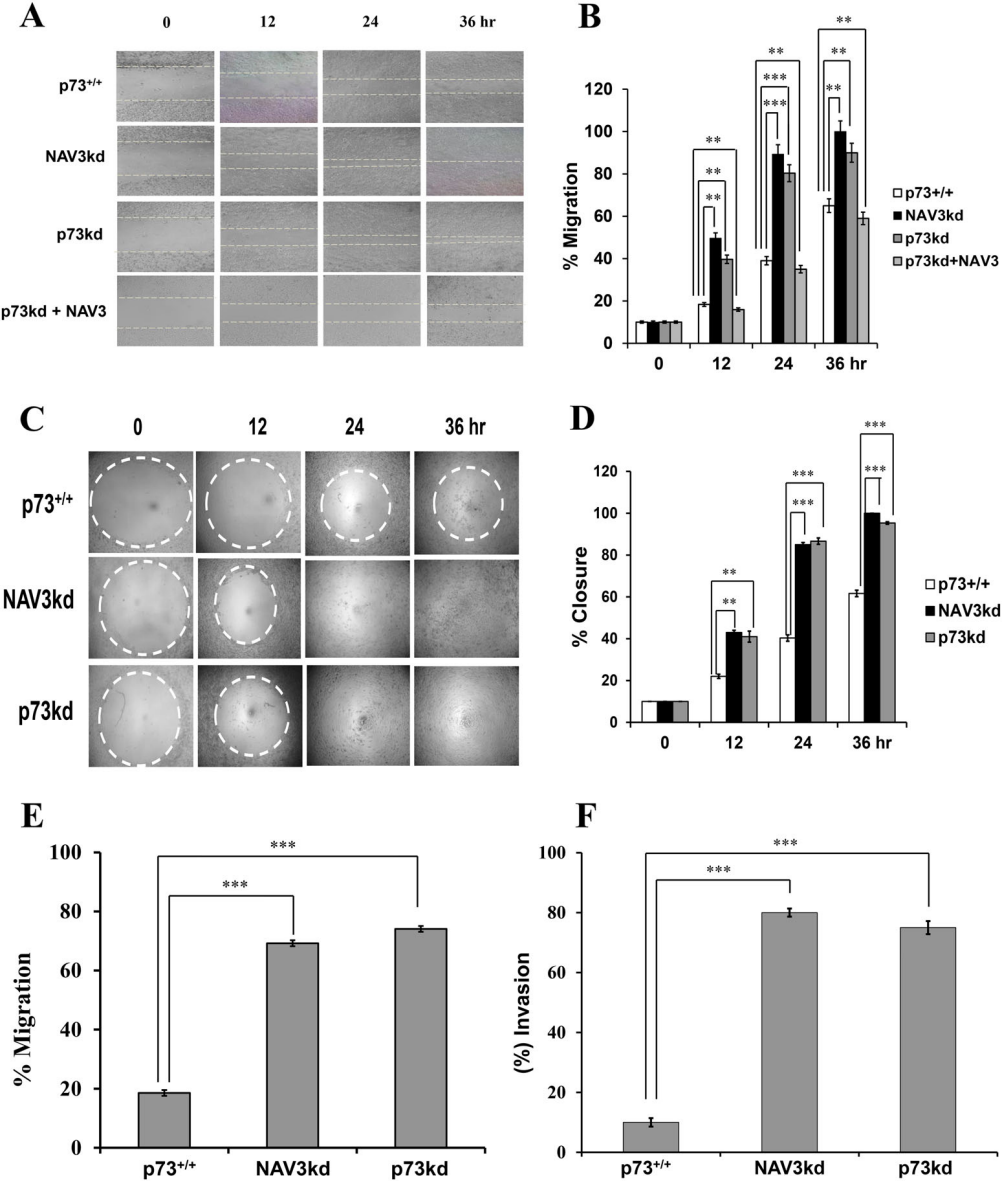
The invasion and migration of colorectal cancer cells were abrogated by NAV3 knockdown. The wound-healing assay (**a**) and Oris cell migration assay (**c**) and their quantification respectively (**b**, **d**) using etoposide (20 μM) treated HCT116p53^−/−^p73^+/+^ (control), HCT116p53^−/−^NAV3kd (NAV3kd), HCT116p53^−/−^p73kd (p73kd) and HCT116p53^−/−^p73kd + NAV3 cells. Imaged every 12 h to calculate the width of each wound (**b**) or width of each radius gel (**d**) using live cell imaging system. The results are presented as mean ± SD (paired two-tailed Student’s *t*-test, **P < 0.01, ***P < 0.001). The migration ability (**e**) and invasion ability (**f**) of etoposide (20 μM) treated HCT116p53^−/−^p73^+/+^ (control), HCT116p53^−/−^p73^+/+^NAV3kd (NAV3kd) and HCT116p53^−/−^p73kd (p73kd) cells using Trevigen’s Cultrex 24 well migration assay (**e**) and Transwell invasion assays (**f**) after 24 h were also determined. Results are presented as the mean ± SD (unpaired two-tailed Student’s *t*-test, ***P < 0.001).

Next, we evaluated the role of NAV3 in p73-mediated suppression of migration and invasion of CRC cells. For this purpose, HCT116p53^−/−^ cells were stably transfected (pooled puromycin-resistant population) with NAV3 shRNA. Cells were then subjected to etoposide treatment (20 μM) for 24 h, followed by RT-qPCR and western blot analysis to measure NAV3 expression. The results confirmed that NAV3 expression was effectively reduced both at mRNA and protein level (Supplementary Figs. S4A and S4B). To evaluate whether NAV3 plays any role in p73 mediated apoptosis, HCT116p53^−/−^p73^+/+,^ p73kd, and NAV3kd cells were treated with etoposide (20 μM) for 24 and 48 h and Annexin V/PI assay was carried out. NAV3kd cells exhibited significant apoptosis similar to p73^+/+^ cells, whereas p73kd cells resulted in highly reduced apoptosis as compared to p73^+/+^ cells, indicating that NAV3 plays no role in p73 mediated apoptosis of cells (Fig. 4). To assess whether NAV3 knockdown affects the migration ability of colorectal cells, wound-healing assay was performed in HCT116p53^−/−^p73^+/+,^ NAV3kd and p73kd cells. Cells were plated and treated with etoposide (20 μM) after 24 h, following which a wound was created and images were taken after every 12 h. Upon genotoxic stress induced by etoposide, the migration of NAV3kd cells and p73kd cells was significantly higher as compared to that of the control cells (Fig. 5a, b). Furthermore, when p73kd cells were transfected with NAV3 overexpression plasmid, the migration of cells reduced as compared to p73kd and NAV3kd cells, indicating that re-expression of NAV3 is sufficient to recapitulate the suppression of the migratory phenotype of the cells (Fig. 5a, b and Supplementary Fig. S4B). To further corroborate this result, we performed an Oris radius migration assay. Results demonstrated that under genotoxic stress NAV3kd cells migrated into the radius gel spot at a much faster rate than that of the control cells and the spot was completely filled within 36 h as compared to that of the control cells. p73kd cells exhibited a similar migration rate as that of NAV3kd cells (Fig. 5c, d). To strengthen this result, we checked the migration ability of NAV3kd and p73kd cells using Trevigen’s Cultrex 24 well migration assay. Cells were plated and treated with etoposide (20 μM) for 24 h. Consistent with the previous results, NAV3kd and p73kd cells demonstrated a significantly higher percentage of migration compared to that of the control cells (Fig. 5e). Next, we evaluated the invasiveness of HCT116p53^−/−^p73^+/+,^ NAV3kd, and p73kd cells using Trevigen’s Cultrex 24 well invasion assay. Cells were plated and treated with etoposide (20 μM) for 24 h. Cells were allowed to invade depending on their invasive potential through membrane pores to the lower chamber for 24 h post etoposide treatment. The NAV3kd cells exhibited nearly 40% increased invasive capability compared to the control cells. As expected, p73kd cells followed a similar trend as that of NAV3kd cells (Fig. 5f). Taken together, these results indicate that NAV3 knockdown can effectively increase cell invasion and migration in a p73-dependent manner. In other words, these results strongly suggest that p73 exert its anti-migratory and anti-invasion role by upregulating NAV3 gene expression.

### NAV3 knockdown promotes EMT transition

**Fig. 6:**
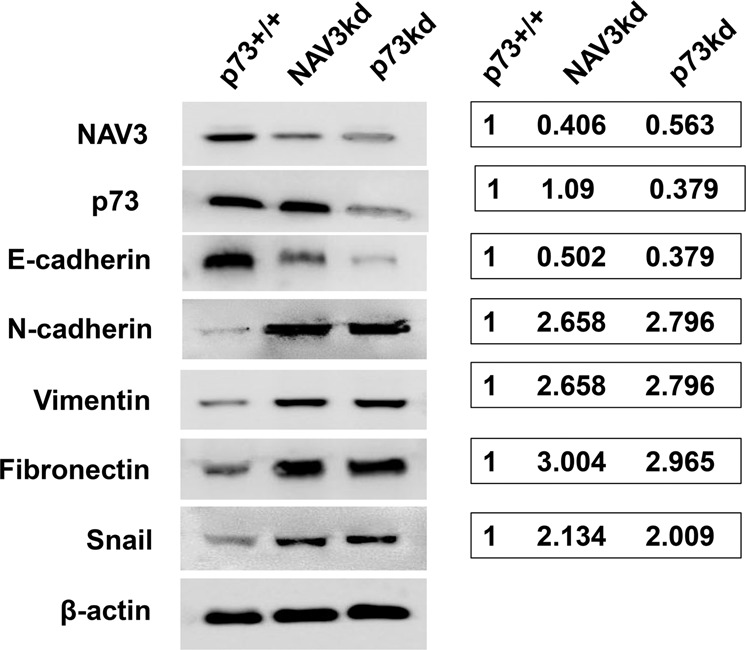
NAV3 knockdown results in altered expression of biomarkers related with invasion and migration. Western blot analysis of different proteins using cell lysate of HCT116p53^−/−^p73^+/+^ cells (control), HCT116p53^−/−^p73^+/+^NAV3kd (NAV3kd) cells and HCT116p53^−/−^p73kd (p73kd) cells treated with etoposide (20 μM) for 24 h. β- actin was used as loading control.

Furthermore, we examined whether NAV3kd has any impact on the expression of biomarkers related with invasion and migration, including EMT transition markers such as E-cadherin, N-cadherin, snail, fibronectin, and vimentin. For this purpose, HCT116p53^−/−^p73^+/+,^ NAV3kd, and p73kd cells were treated with etoposide (20 μM) for 24 h and western blot analysis was performed. Results demonstrated that NAV3 knockdown significantly downregulated protein expression of E-cadherin as compared to the control cells. Meanwhile, protein expression of the other markers such as—snail, vimentin, N-cadherin, and fibronectin were significantly upregulated in NAV3kd cells as compared to that of the control cells. Similarly, knockdown of endogenous p73 decreased E-cadherin expression and increased N-cadherin, snail, vimentin, and fibronectin expression (Fig. 6). Taken together, these results confirm that NAV3 restricts the migration and invasion potential of CRC cells and plays an important role in suppression of EMT in a p73-dependent manner.

### Knockdown of NAV3 causes an upregulation of MMP2, MMP9, and actin-binding protein Cortactin

**Fig. 7:**
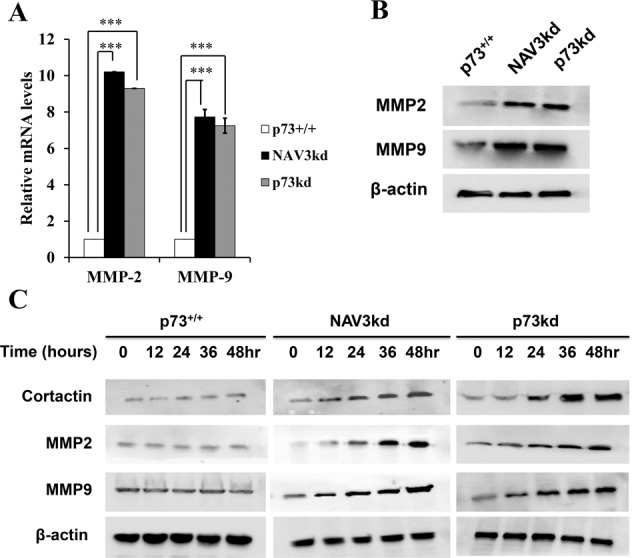
NAV3 knockdown results in an increase in Cortactin, MMP2 and MMP9 levels. Expression of MMP2 and MMP9 at RNA level by RT-qPCR (**a**) and protein level by western (**b**) in HCT116p53^−/−^p73^+/+^ (control), HCT116p53^−/−^p73^+/+^NAV3kd (NAV3kd) and HCT116p53^−/−^p73kd (p73kd) cells treated with etoposide (20 μM) for 24 h (paired two-tailed Student’s *t*-test, ***P < 0.001). β-actin was used for normalization. The results of three independent experiments are presented as the mean ± SD. **c** Western to check the expression of Cortactin, MMP2 and MMP9 as a function of time of etoposide (20 μM) treatment in HCT116p53^−/−^p73^+/+^ (control), HCT116p53^−/−^p73^+/+^NAV3kd (NAV3kd) and HCT116p53^−/−^p73kd (p73kd) cells. β-actin was used as loading control.

As NAV3kd increases the migration and invasive potential of CRC cells, next we wanted to check the expression of matrix metalloproteinases (MMPs)—MMP2 and MMP9 in the presence and absence of NAV3. Studies have revealed that MMP2 and MMP9 promote the degradation of the extracellular matrix, proliferation, and invasion of colon cancer cells^27,28^. In our study (Fig. 6), NAV3kd resulted in upregulation of Snail, an important EMT marker, which has been reported to significantly increase MMP2 expression^29^. Hence, HCT116p53^−/−^p73^+/+,^ NAV3kd, and p73kd cells were treated with etoposide (20 μM) for 24 h, followed by RT-qPCR and western blot analysis experiments to check the expression levels of MMP2 and MMP9. As expected, we observed an upregulation of MMP2 and MMP9 in NAV3kd and p73kd cells both at mRNA (Fig. 7a) and protein level (Fig. 7b). Furthermore, previous studies have established that the secretion of MMP2 and MMP9 is dependent on the levels of cortactin expression^30^. Cortactin is an actin related protein 2/3 complex-activating and filamentous (F)-actin-binding protein that is implicated in tumor cell motility and metastasis^31^. Hence, we further decided to check the kinetics of cortactin, MMP2 and MMP9 expression upon etoposide treatment (20 μM) in control, NAV3kd and p73kd cells at various time points from 12 to 48 h. Cortactin protein expression was found to be enhanced upon genotoxic stress in a time-dependent manner in NAV3kd and p73kd cells as compared to control cells (Fig. 7c). A similar trend was observed in the expression levels of MMP2 and MMP9 in a time-dependent manner post genotoxic stress in NAV3kd and p73kd cells. Taken together, these results suggest that NAV3 might regulate cortactin, which in turn increases the expression of MMP2 and MMP9.

### IHC analysis of NAV3 and p73 expression levels in non-metastatic and metastatic colon cancer tissues

**Fig. 8:**
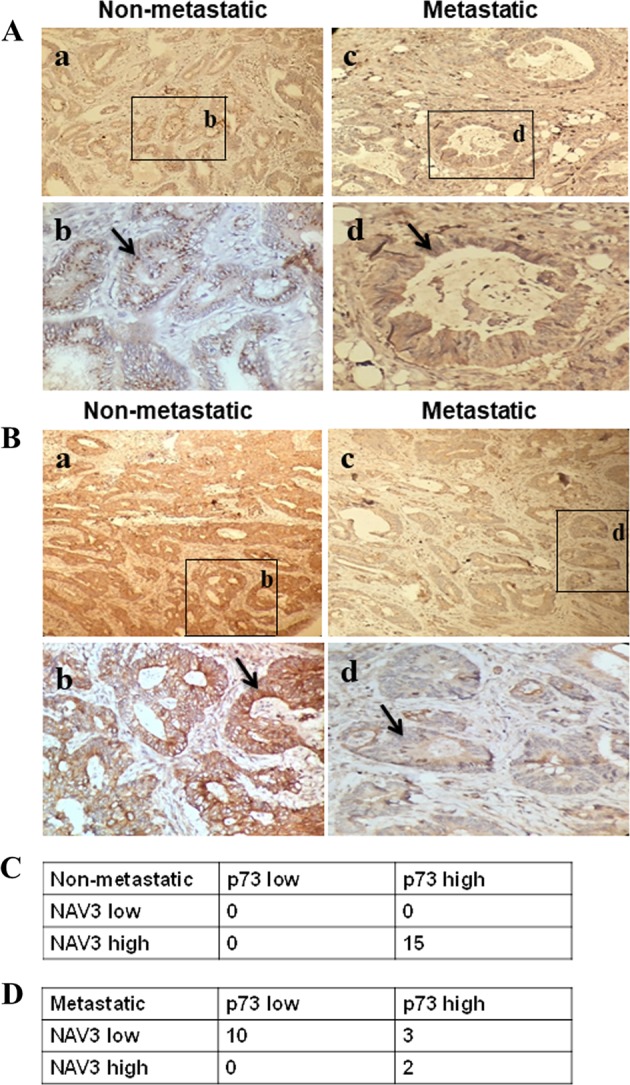
Altered expression of NAV3 and p73 in metastatic colon cancer tissue. Fifteen samples of human non-metastatic and 15 samples of human metastatic colon cancer tissues were evaluated for NAV3 and p73 expression by Immunohistochemistry. **a**, **b** Representative photographs of immunohistochemistry (**a**) for p73 and (**b**) NAV3 expression in metastatic and non-metastatic colon carcinoma tissue. (magnification: ×400 for the inserts—**b** and **d**; X200 for all others—**a** and **c**). See also Supplementary Fig. S5A and S5B. **c** Association study between p73 and NAV3 expression in non-metastatic CRC tissue samples. **d** Association study between p73 and NAV3 expression in metastatic CRC tissue samples.

To confirm the positive correlation between NAV3 and p73, 15 samples of human non-metastatic and 15 samples of human metastatic colon cancer tissues were evaluated for NAV3 and p73 expression by IHC (Supplementary Table S1). The p73 protein is localised mainly in the nuclei of the cancer cells while NAV3 is expressed mostly in the cytoplasm of the cancer cells. p73 expression level was significantly upregulated in non-metastatic CRC tissue samples as compared to metastatic colon cancer tissue samples (Fig. 8a-compare a and c, b, and d, Supplementary Fig. S5A). Similarly, NAV3 expression was significantly high in non-metastatic CRC tissue samples as compared to metastatic tissue samples (Fig. 8b-compare a and c, b and d, Supplementary Fig. S5B). Association studies were also performed to determine the correlation between p73 and NAV3 expression in non-metastatic and metastatic tissue samples respectively (Fig. 8c, d). We performed scoring of expression level on a scale of 1 to 4 based on the intensity of staining. 15/15 cases of non-metastatic CRC showed high intensity (2 or 3) for both NAV3 and p73 expression. In contrast, 10/15 cases of metastatic CRC showed low intensity (0 or 1) for both NAV3 and p73 expression. Based on our results on p73kd and NAV3kd cell lines and IHC, we envisage that this significant downregulation of p73 and NAV3 protein levels could promote metastasis in colon cancer.

## Discussion

Navigator-3 (NAV3), a microtubule-binding protein, has previously been implicated in axonal guidance^32,33^. While seldom mutated in cancer, the range of tumors in which aberrant NAV3 expression was encountered is extensive: colorectal, breast, melanoma, adrenal carcinoma, glioblastoma and neuroblastoma^34–36^. It has been reported that NAV3 copy number changes promote early carcinogenesis by upregulating IL23R and GnRGR expression, catering to a microenvironment of inflammation, and supplying malignant cells with a growth advantage^24^. Another study implicated the role of NAV3 in inhibiting breast cancer progression by regulating microtubule dynamics. In the presence of growth factors, NAV3 accumulates at the plus ends of microtubules and amplifies their polarized growth, while NAV3 depletion pruned microtubule growth and enriched random cell migration^22^. However, the exact pathway by which NAV3 aberrations aid metastasis and tumor progression in patients and in animal models remains to be elucidated. Similarly, p73 has a well-established role as a tumor suppressor and controls cell invasion, migration, metastasis and EMT-mediated cancer cell stemness by regulation of POSTN^37^, FOXF1^38^, and p57Kip2^19^ in a variety of cancer cells. However, its role in CRC invasion and metastasis is poorly understood. In line with these studies, our study identifies NAV3 as a novel downstream target of p73 and reveals that p73 mediates its anti-metastatic function through upregulation of NAV3 expression.

Our study delineates the molecular mechanisms underlying the pivotal role of p73 in suppression of metastasis through modulation of NAV3. Discovery of two putative p73-binding sites in the NAV3 promoter led us to explore whether p73 regulates NAV3 expression. Treatment of HCT116p53^−/−^p73^+/+^ cells with etoposide, activated endogenous p73, with a concomitant increase in the expression of NAV3 both at mRNA and protein levels. Consistent with these observations, over-expression of p73 also upregulated NAV3 expression in HCT116p53^−/−^p73^+/+^ cells. Additionally, luciferase assays revealed that the putative p73 binding sites in NAV promoter are functional and activate transcription in a p73-dependent manner. Importantly, corresponding constructs that either lacked the p73-binding site or that contained mutations within the putative site exhibited no p73-dependent transcriptional activation of reporter gene, suggesting that the predicted p73 binding sites are indeed essential for p73-mediated transactivation of NAV3. The binding of p73 to the promoter region of NAV3 under in vivo conditions was unequivocally established by ChIP assays.

The functional significance of regulation of NAV3 by p73 was established by carrying out wound —healing assays and Transwell migration and invasion analysis. These experiments showed that NAV3 knockdown increased colon cancer cell migration and invasiveness in a p73-dependent manner. Importantly, NAV3 works downstream of p73 in specifically regulating cell migration as overexpression of NAV3 in p73kd cells could suppress migration (see Fig. 5a, b). However, NAV3 has no role to play in p73-mediated regulation of apoptosis (see Fig. 4). A very early event in which cancer cells switch to an invasive and aggressive phenotype is known as epithelial-mesenchymal transition (EMT)^39–41^. A widely accepted marker for EMT is loss of E-cadherin^42^ and upregulation of N-cadherin^43^, vimentin^44^, snail^45^, and fibronectin^46^. Using NAV3kd cells and p73kd cells, we demonstrated a significant downregulation of E-cadherin, and significant upregulation of the mesenchymal markers, compared to that of the controls, thus suggesting that NAV3 plays an important role as a metastasis inhibitor in a p73-dependent manner. Taken together, these results strongly implicate that p73 exhibits its anti-metastatic function through upregulation of NAV3.

MMPs play an important role in tumor invasion and metastasis, and among many MMPs, MMP2 and MMP9 are well characterized in invasiveness and metastasis^27,28^. Once we established a role of NAV3 in the change in expression of EMT markers, we then explored the effect of NAV3 knockdown on MMP2 and MMP9 expression in HCT116p53^−/−^p73^+/+^ cells. RT-qPCR and western blot analysis revealed a significant increase in MMP2 and MMP9 expression in NAV3kd and p73kd cells. Since cortactin, an actin-binding protein, is known to regulate MMP2 and MMP9 secretion and extracellular matrix degradation^30^, we further checked the levels of cortactin in NAV3kd and p73kd cells and found them to be upregulated at both mRNA and protein levels. It will be interesting to further explore how exactly NAV3 causes upregulation of Cortactin.

A recent study assessed the expression of p73 gene in tumor-surgical margin specimens of CRC patients at the mRNA and protein levels and found decreased expression of p73 in CRC tumor tissue as compared to unchanged colon mucosa^16^. In line with these results, we wanted to compare the expression of NAV3 and p73 by IHC in metastatic and non-metastatic colon cancer tissues and determine whether there is co-expression of NAV3 and p73 as implicated in our studies. Importantly, we observed decreased expression of both NAV3 and p73 in metastatic tissues as compared to that of non-metastatic tissues, further corroborating our studies. Moreover, highly significant association between NAV3 levels and p73 levels was observed in both non-metastatic and metastatic tumor tissue samples. Taken together, our study provides conclusive evidence that Navigator-3 is a direct transcriptional target of p73 and plays crucial role in response to genotoxic stress in p73-mediated inhibition of cancer cell invasion, migration and metastasis. As p53 or it’s associated pathways are frequently non-functional in cancer, this study will prove to be a big leap towards precision medicine to fight against metastatic cancer cells harboring mutated or non-functional p53.

## Materials and methods

### Tissue samples

Thirty cases of paraffin-embedded biopsy proven colorectal carcinoma tissues were selected from the archives of National Institute of Pathology, Safdarjung Hospital, New Delhi, to study the expression of p73 and Navigator-3 (NAV3) by IHC. As there was no previous report, we chose 30 clinical samples for preliminary experiment by convention. The inclusion criteria was retrospective metastatic and non-metastatic biopsy samples. The knowledge of the metastatic and non-metastatic samples was restricted to the clinician and the results were compared only after IHC was performed. The study was approved by the Ethical Review Board of National Institute of Pathology, Safdarjung Hospital (Reference number: NIP-IEC/14-06-18/03) and Dr.B.R.Ambedkar Center for Biomedical Research, University of Delhi (Reference number: ACBR/IHEC/DS-03/09-18).

### Cell lines, culture conditions, and transfection

Cell lines HCT116p53^−/−^ and HCT116p53^+/+^ were obtained from the lab of Bert Vogelstein, Johns Hopkins University, Maryland, US and cultured in Dulbecco Modified Eagle’s Medium (DMEM) containing fetal bovine serum (Invitrogen) and 100 U/ml Penicillin Streptomycin at 37 °C in humidified air with 5% CO_2_. H1299p53^−/−^ cell line was obtained from the lab of Kulbushan Sharma, Institute of Nuclear Medicine and Allied Sciences, New Delhi, India. They were regularly tested for mycoplasma contamination. HCT116p53^−/−^p73kd cell line was generated by transfecting pBABEU6 vector containing shRNA targeting p73 (pooled puromycin-resistant population). HCT116p53^−/−^NAV3kd cell line was generated by transfecting pBABEU6 vector containing shRNA targeting NAV3 (pooled puromycin-resistant population). HCT116p53^+/+^p73kd cells were generated by transfecting pBABEU6 vector containing shRNA targeting p73 (pooled puromycin-resistant population). Transfections were carried out using Lipofectamine 2000 (Invitrogen) according to manufacturer’s protocol. For drug treatment, cells were grown to ~50% confluency before exposure to etoposide (Sigma) for the indicated time.

### Plasmids and shRNA

For luciferase assay, the NAV3 promoter region U1 (nucleotides −2269/−1437 from the TSS), region U2 (nucleotides −1456/−341 from the TSS), region D1 (nucleotides +8 /+1101 from the TSS) and region D2 (nucleotides +1958/+2638 from the TSS) was cloned into the pGL4.20 vector (Promega). The primers and shRNA used for cloning are listed in Supplementary Table S2. NAV3 overexpression plasmids were a kind gift from Prof. Yosef Yarden, Weizmann Institute of Science, Israel.

### Luciferase assays

For transient transfection, cells were seeded in a 12 well plate and grown overnight. 500 ng of the different NAV3 promoter constructs were co-transfected with 500 ng of Renilla luciferase construct (pRL-TK) using Lipofectamine 2000 reagent (Thermo Fischer Scientific) according to the manufacturer’s recommendations. Etoposide (20 μM) was added 6 h post transfection. After 24 h incubation, cells were washed twice with PBS and lysed in passive lysis buffer (300 μl/well) provided by the Dual-Luciferase Reporter Assay System (Promega). Firefly and Renilla luciferase activities were measured from the lysate using luminometer. Firefly luciferase values were normalised to Renilla luciferase. Error bars are mean ± SD of three independent experiments.

### ChIP assays

ChIP assays were performed manually. Briefly, HCT116p53^−/−^ and HCT116p53^−/−^p73kd cells treated with etoposide (20 μM) for 24 h were cross-linked with 1% formaldehyde for 10 min, treated with 10X Glycine for 5 min, washed with PBS and lysed in ChIP lysis buffer. The lysate was then sonicated using QSonicator to shear the DNA into fragments of ~200–1000 bp. The samples were precleared with Protein A agarose/Salmon sperm DNA slurry. Control IgG or anti-p73 antibodies were added and incubated overnight at 4 °C followed by incubation with fresh Protein A/ Salmon sperm DNA slurry for 1 h. Before immunoprecipitation, 1% of the supernatant was removed as “input”. Precipitated chromatin complexes were removed from the beads through 30 min incubation with 200 μl of elution buffer (20% SDS, 1 M NaHCO_3_). Finally, the protein-DNA cross-links were reversed by incubation with 5 M NaCl at 65 °C for 4–5 h and immunoprecipitated DNA was analysed by qPCR using primers flanking the p73 binding site BS1; BSS1 (−1257 to −1042) present in U2 region of NAV3 promoter and primers flanking the p73 binding site BS2; BSS2 (+2396 to +2590) present in D2 region of NAV3 promoter. Primer sequences are listed in the Supplementary Table S2.

### Site-directed mutagenesis

Specific nucleotides were mutated using Q5 Site-Directed Mutagenesis Kit (New England Biolabs). Primers containing mutated nucleotides were designed using NEBaseChanger tool and exponential amplification was carried out using the designed primers and master mix formulation of Q5 Hot Start High-Fidelity DNA polymerase. After PCR, the amplified material was added directly to a unique Kinase-Ligase-Dpn1 (KLD) enzyme mix and incubated at room temperature for 5 min. Following this, transformation into high-efficiency NEB 5-alpha competent *E.coli* cells was carried out. Site-directed mutagenesis was confirmed through DNA sequencing (Pragati Biomedicals).

### Annexin-V and propidium Iodide staining

HCT116p53^−/−^p73^+/+^, HCT116p53^−/−^NAV3kd and HCT116p53^−/−^p73kd cells were treated with etoposide (20 μM) for 24 and 48 h. They were then stained with APC (allophycocyanin) labeled annexin-V and PI as per the manufacturer’s guidelines (eBiosciences, USA). Population was then analysed for percentage of cells in healthy, early apoptotic and late apoptotic phase on FACScalibur using CellQuestPro software (Becton Dickinson, USA).

### Western blotting analysis

Cells were lysed in lysis buffer (1 M Tris-HCl pH 8, 5 M NaCl, 0.5 M EDTA, 3% Na_4_P_2_O_7_, 10% NP40, 1 M NaF, 200 mM phenyl methylsulphonyl fluoride, 1X Protease inhibitor cocktail; Roche, Basel, Switzerland). Pierce BCA Protein Assay Kit (Thermo Fisher Scientific) was used to determine the sample concentration. Bovine Albumin Serum (BSA, Invitrogen) was used to create a standard curve for protein concentration and for normalizing the concentration among samples. Equal amounts of proteins per sample was subjected to SDS-PAGE and transferred to a PVDF membrane (Millipore). The antibody of interest was incubated at 4 °C overnight in 5% BSA solution or 5% skimmed milk solution. The blots were then incubated with HRP-conjugated secondary antibodies (Santacruz) at room temperature for 45 min, followed by ECL-based detection (Bio-Rad).

### Migration assay using calcein-AM

For migration assays, cells were serum starved 24 h prior to assay. Cells were then centrifuged at 250 × *g* for 10 min, supernatant removed, washed with 1× wash buffer, resuspended at 1 × 10^6^ cells/ml in a serum free medium. Cells (0.1 × 10^6^ cell/ insert) were plated in the upper compartment of Trevigen’s Cultrex 24 well cell migration plate, which utilizes a simplified Boyden chamber design with an 8 μm pore size polyethylene terephthalate (PET) membrane. Five hundred microliters of complete media (FBS added) was added to the bottom chamber, the whole apparatus was assembled and incubated at 37 °C in a CO_2_ incubator for 36 h. After 36 h, the top and the bottom chambers were aspirated and washed with 500 μl of 1X wash buffer. Detection of cell migration was quantified using Calcein-AM. 500 μl of Cell Dissociation Solution/Calcein-AM was added to the lower chamber and the plate was read at 485 nm excitation, 520 nm emission.

### Invasion assay using calcein-AM

The cell’s invasive ability was measured in Trevigen’s Culture Coat 24 well BME (Basement membrane Extract) coated Cell Invasion Chambers. 10% FBS was added to the bottom chamber as a chemo-attractant. Cells were serum-starved 24 h prior to the assay. Cells (0.1 × 10^6^ cells/ insert) were seeded and etoposide (20 μM) was added and incubated for 36 h before analysis. Non-migrating cells on the upper side of the membrane were removed, and the cells that migrated to the lower chamber were quantified using Calcein-AM added in Cell Dissociation Solution. The number of invading cells was determined by reading the plate at 485 nm excitation, 520 nm emission.

### Wound-healing assay

Cells were seeded into 24 well plates using 1% FBS-containing culture media with or without etoposide. Twenty-four hours after plating, a wound was created using a cell scratcher, washed and replaced with media containing etoposide. The culture plates were marked as reference points close to the scratch. Images of the scratched area at the reference points were recorded immediately after the scratch and then at 12, 24, and 36 h using a phase-contrast microscope at ×10 magnification. The distances of the scratched area were determined and measured using ImageJ software. The average migration distance of each well was calculated. The area of wound was quantified by Java’s Image J software (http://rsb.info.nih.gov) using the polygon selection mode. The migration of cells toward the wounds was expressed as percentage of wound closure:

$${\mathrm{\% }}\,{\mathrm{of}}\,{\mathrm{wound}}\,{\mathrm{closure}} = [({\mathrm{At}} = 0\,{\mathrm{h}}-{\mathrm{At}} = \Delta \,{\mathrm{h}})/{\mathrm{At}} = 0\,{\mathrm{h}}] \times 100\% ,$$

where At = 0 h is the area of wound measured immediately after scratching, and At = Δh is the area of wound measured 12 or 24 h after scratching.

### Oris radius cell migration assay

Cells were seeded in a 96-well plate with “stopper” barriers that create a central cell-free detection zone for cell migration experiments. Removing the stoppers allows the cells to migrate into the detection zone at the center of each well. The cells were then treated with etoposide (20 μM). Images were taken at 12, 24, and 36 h using a phase-contrast microscope at ×10 magnification. The area of the radius gel was quantified by Java’s Image J software (http://rsb.info.nih.gov) using the polygon selection mode. The migration of cells toward the radius gel was expressed as percentage of gel closure:

$${\mathrm{\% }}\,{\mathrm{of}}\,{\mathrm{closure}} = [({\mathrm{At}} = 0\,{\mathrm{h}}-{\mathrm{At}} = \Delta \,{\mathrm{h}})/{\mathrm{At}} = 0\,{\mathrm{h}}] \times 100\% ,$$

where At = 0 h is the area of radius gel measured immediately after seeding, and At = Δh is the area of radius gel measured 12 or 24 h after etoposide treatment.

### RNA extraction and real-time qRT-PCR

Total RNA was extracted from cells using Qiagen’s RNA extraction kit. cDNA synthesis was carried out using cDNA synthesis kit (Applied Biosystems) following the manufacturer’s protocol. Quantitative PCR (qPCR) was then carried out for the respective genes using validated qPCR primers (IDT) and SYBR Green mastermix (Promega) in BioRad S-1000 Thermo cycler. β-actin was used as internal control for all samples. Primers used are listed in Key Resources Table. The ΔΔCt method was used to calculate the relative abundance of RNA for each gene compared with β-actin expression. Error bars are mean ± SD of three independent experiments with triplicate samples.

### Immunohistochemistry (IHC)

Five-μm tissue sections were deparaffinised, rehydrated in graded alcohols, and processed using the Master-polymer plus peroxidase method. Briefly, the sections were submitted to antigen retrieval by microwave oven treatment for 20 min in 10 mM citrate buffer (pH 6). Slides were subsequently incubated with peroxidase block for 10 min and then overnight incubation at 4 °C with the appropriately diluted primary antibody. The rabbit anti-p73 polyclonal antibodies (~100 μg/μl) were used at a 1:50 dilution and the rabbit anti-NAV3 antibodies (~100 μg/μl) were used at a 1:50 dilution. After the primary antibody treatment, samples were incubated with primary antibody amplifier for 30 min, followed by HRP labeled secondary antibody for 30 min. 3-3′-Diaminobenzidin was used as the chromogen and hemotoxylin and eosin as the counterstain. Results were scored by estimating the percentage of tumor cells showing characteristic nuclear staining and cytoplasmic staining. Immunoreactivity for p73 and NAV3 in non-metastatic and metastatic CRC tissues was scored as follows: None (<5%, score 0), Weak (+, 5%–25%, score 1), Moderate (++, 25%–75%, score 2), Intense (+++, >75%, score 3).

### Statistics

All statistical analyses were performed using Prism software (Graphpad Prism 5). Values were expressed as mean ± (SD) for parametric data. Significance was determined by two-tailed Student’s *t*–test. For all analyses *P* < 0.05 was considered statistically significant, and *P < 0.05, **P < 0.01, ***P < 0.001. The statistical significance of the expression of p73 and NAV3 were calculated by Pearson *χ*² test.

## Supplementary information

Supplementary Information
