# Supplementary material for: p73 – NAV3 axis plays a critical role in suppression of colon cancer metastasis
Source: Oncogenesis. 2020 Feb 6;9(2):12. doi: 10.1038/s41389-020-0193-4 (PMC7005187; doi:10.1038/s41389-020-0193-4)
Supplement: Supplementary file 1 — Supplementary Information [file 41389_2020_193_MOESM1_ESM.docx]

**Figure S1. Analysis of genotoxic stress mediated up-regulation of p73 and NAV3. A)**

HCT116p53^-/-^ (control) cells were treated with etoposide (20 μM) and p73 plasmid for 24

hours. Cell lysates were prepared and western blotting was performed for NAV3 and p73. β-

actin was used as a loading control. **B)** HCT116p53^-/-^ and H1299p53^-/-^ cells were treated with etoposide (20 μM) for 24 and 48 hours. Cell lysates were prepared and western blotting was performed for NAV3 and p73. β-actin was used as a loading control. **C)** HCT116p53^-/-^ (control) cells were stably transfected (pooled puromycin-resistant population) with p73 shRNA (p73kd). Cell lysates were prepared post 24 hours etoposide treatment and western blot analysis was performed for p73 protein. β-actin was used as loading control. RT-qPCR for NAV3 **(D)** and western **(E)** for NAV3, p73 and β-actin (loading control) in etoposide treated (20 μM) HCT116p53^-/-^p73^+/+^ (control) and HCT116p53^-/-^p73kd (p73kd) cells for the indicated time points was done. For **(D)**, paired two-tailed Student’s t-test, *P<0.05, **P<0.01, ***P<0.001 was used. The results of three independent experiments are presented as the mean + SD. **F)** RT-qPCR for NAV3 in HCT116p53^+/+^ (control) and HCT116p53^+/+^p73kd (p73kd) cells post 24 hours etoposide treatment. Fold changes were calculated after normalization with β-actin. The results of three independent experiments are presented as the mean + SD (unpaired two-tailed Student’s t-test, ***P<0.001).**G)** HCT116p53^+/+^p73^+/+^ (control) and HCT116p53^+/+^p73kd cells (p73kd) were treated with etoposide (20 μM) for 24 hours. Cell lysates were prepared and NAV3 expression was checked by western blotting. β-actin was used as loading control.

**Figure S2. Identification of p73 binding sites in the promoter of NAV3 gene**. **A)** Structure

of putative p73 DNA binding element. **B)** Schematic representation of 11 putative p73 half

binding sites in the upstream region (1949 bp) and 9 putative half binding sites in the

downstream region (2630 bp) relative to the TSS using TFBind software and JASPAR

database. **C)** Table showing description of regions U1, U2, D1 and D2.

**Figure S3. p73 targets NAV3 promoter. A)** Luciferase assay in untreated and etoposide (20 μM, 48 hours) treated H1299p53^-/-^p73^+/+^ (control) cells transfected with empty pGL4.2 luciferase vector, pGL4.2-NAV3 promoter-reporter constructs and pRLT-K loading control. The results are presented as mean + SD of three independent experiments (unpaired two-tailed Student’s t-test, **P<0.01, ***P<0.001). (**B and C)** ChIP RT-qPCR performed in etoposide (20 μM) treated HCT116p53^-/-^p73^+/+^ (control) and HCT116p53^-/-^p73kd (p73kd) cells for the indicated time points with antibody against p73 and primers specific to BSS1 region (-1257 to -1042) containing BS1 binding site present in U2 region **(B)** and BSS2 region (+2396 to +2590) containing BS2 binding site present in D2 region **(C)** of NAV3 promoter. The results are presented as mean + SD of three independent experiments (paired two-tailed Student’s t-test, *P<0.05, **P<0.01, ***P<0.001).

**Figure S4. Increase in the migration and invasion capacity of colon cancer cells by**

**knockdown of NAV3. A)** HCT116p53^-/-^ p73^+/+^ (control) cells were stably transfected (pooled

puromycin-resistant population) with NAV3 shRNA. Following transfection, the cells were

subjected to etoposide treatment (20 μM) for 24 hours. RNA was isolated and qRT-PCR was

performed to check NAV3 mRNA level. Unpaired two-tailed Student’s t-test was used;

***P<0.001. **B)** HCT116p53^-/-^p73^+/+^ (control), HCT116p53^-/-^p73^+/+^NAV3kd (NAV3kd) and HCT116p53^-/-^p73kd (p73kd) cells were subjected to etoposide treatment (20 μM) for 24 hours. Cell lysates were prepared and western blot analysis was performed for the indicated proteins. β- actin was used as loading control. HCT116p53^-/-^p73kd were transfected with NAV3 overexpression plasmid for 24 hours. Following transfection, the cell lysates were prepared for western blotting and p73 and NAV3 protein levels were checked and compared with etoposide (20 μM, 24 hours) treated HCT116p53^-/-^p73^+/+^ (p73^+/+^), p73kd and NAV3kd cells.

**Figure S5. Analysis of p73 and NAV3 expression in metastatic and non-metastatic CRC samples by Immuno-Histochemistry.** 15 samples of human non-metastatic and 15 samples of human metastatic colon cancer tissues were evaluated for NAV3 and p73 expression by Immuno-histochemistry.  **A)** p73 expression level was higher (Intensity score 2 and 3) in non-metastatic CRC tissue samples than that in metastatic CRC tissue samples (P<0.001, χ² test). **B)** NAV3 expression level was up-regulated (Intensity score 2 and 3) in non-metastatic CRC tissue samples as compared to metastatic CRC tissue samples (P < 0.001, χ² test).

**Table S1: Expression score of p73 and NAV3 protein in non-metastatic and metastatic colon carcinoma tissue samples (+++ (3) very high, ++ (2) medium, + (1) low, 0 none).**

| **Non-Metastatic Cases** | **p73 (SCORE)** | **NAV3 (SCORE)** | **Metastatic cases** | **p73 (SCORE)** | **NAV3 (SCORE)** |
| --- | --- | --- | --- | --- | --- |
| CASE 1 | +++ (3) | +++ (3) | CASE 16 | + (1) | + (1) |
| CASE 2 | +++ (3) | +++ (3) | CASE 17 | + (1) | (0) |
| CASE 3 | ++ (2) | +++ (3) | CASE 18 | + (1) | + (1) |
| CASE 4 | +++ (3) | +++ (3) | CASE 19 | ++ (2) | + (1) |
| CASE 5 | +++ (3) | ++ (2) | CASE 20 | ++ (2) | + (1) |
| CASE 6 | +++ (3) | +++ (3) | CASE 21 | + (1) | + (1) |
| CASE 7 | +++ (3) | +++ (3) | CASE 22 | ++ (2) | ++ (2) |
| CASE 8 | ++ (2) | ++ (2) | CASE 23 | + (1) | + (1) |
| CASE 9 | ++ (2) | ++ (2) | CASE 24 | ++ (2) | ++ (2) |
| CASE 10 | +++ (3) | +++ (3) | CASE 25 | + (1) | + (1) |
| CASE 11 | +++ (3) | +++ (3) | CASE 26 | + (1) | + (1) |
| CASE 12 | ++ (2) | +++ (3) | CASE 27 | + (1) | + (1) |
| CASE 13 | ++ (2) | +++ (3) | CASE 28 | + (1) | + (1) |
| CASE 14 | +++ (3) | +++ (3) | CASE 29 | ++ (2) | + (1) |
| CASE 15 | ++ (2) | ++ (2) | CASE 30 | + (1) | + (1) |

**Table S2:** **Reagents and** **primer sequences used in the study:**

| REAGENT or RESOURCE SOURCE IDENTIFIER | | | | | |
| --- | --- | --- | --- | --- | --- |
| Antibodies | | | | | |
| anti-p73 | | Santacruz | | | Cat# sc-7957; RRID:AB_2207314 |
| anti-p73 | | Abcam | Cat# ab14430; RRID:AB_301211 | | |
| anti-NAV3 | Sigma | | | | Cat#HPA032111; RRID:10603881 |
| anti-β-actin | | Santacruz | | Cat# sc-47778; RRID:AB_626632 | |
| anti-fibronectin | | Santacruz | | Cat# sc-8422; RRID:AB_627598 | |
| anti-E-cadherin | | Abcam | | Cat# ab40772; RRID:AB_731493 | |
| anti-SNAIL | | Abcam | | Cat# ab85936; RRID:AB_1925448 | |
| anti-cortactin | | Abcam | | Cat# ab81208; RRID:AB_1640383 | |
| anti-MMP2 | | Abcam | | Cat# ab37150; RRID:AB_881512 | |
| anti-MMP9 | | Abcam | | Cat# ab38898; RRID:AB_776512 | |
| anti-N-cadherin | | Abcam | | Cat# ab76011; RRID:AB_1310479 | |
| anti-vimentin | | Abcam | | Cat#ab92547; RRID:AB_10562134 | |
| Oligonucleotides | |  | |  | |
| Real-Time Primers:  NAV3 FP:5′ GGGGTACCTGACCAGCAGTGTTCTTGT  TG 3′ | | IDT | | N/A | |
| NAV3 RP:5′ CCGCTCGAGACCACTGTTGAAATCTCC  CCT 3′ | | IDT | | N/A | |
| p73 FP:5′ GCACCACGTTTGAGCACCTCT 3′ | | IDT | | N/A | |
| p73 RP:5′ GCAGATTGAACTGGGCCATGA 3′ | | IDT | | N/A | |
| MMP2 FP:5′ TCTCCTGACATTGACCTTGGC 3′ | | IDT | | N/A | |
| MMP2 RP;5′ CAAGGTGCTGGCTGAGTAGATC 3′ | | IDT | | N/A | |
| MMP9 FP:5′ TTGACAGCGACAAGAAGTGG 3′ | | IDT | | N/A | |
| MMP9 RP:5′ GCCATTCACGTCGTCCTTAT 3′ | | IDT | | N/A | |
| Cortactin FP:5′ TGAGTGTGTGTTCTTCCCCAAG 3′ | | IDT | | N/A | |
| Cortactin RP:5′ CACGTGACCTTCTGGAAAGACA 3′ | | IDT | | N/A | |
| β-actin FP:5′ CCCTGGACTTCGAGCAAGAGAT 3′ | | IDT | | N/A | |
| β-actin RP:5′ AAGGTAGTTTCGTGGATGCCACA 3′ | | IDT | | N/A | |
| p73 shRNA:  FP:5′ TCGAGGTCCGCCAAGGGTTACAGAGCATTT  ATTCAAGAGATAAATGCTCTGTAACCCTTGGCGGA  CC 3′ | | IDT | | N/A | |
| RP:5′ GATCCAAAAAGCCAAGGGTTACAGAGCATTT  ATCTCTTGAATAAATGCTCTGTAACCCTTGGCGGA  CC 3′ | | IDT | | N/A | |
| NAV3 shRNA;  FP:5′ TCGAGGTCCGCCAGTCCTTATCTAAGCCTCTT  CAAGAGAGAGGCTTAGATAAGGACTGGCTTTTTG 3′ | | IDT | | N/A | |
| RP:5′ CCAGGCGGTCAGGAATAGATTCGGAGAAGT  TCTCTCTCCGAATCTATTCCTGACCGAAAAACCTA  GTCGA 3′ | | IDT | | N/A | |
| Primer: U1 region  FP:5′ GGGGTACCGCAAAGAAGCCCTCAGTTTG 3′ | | IDT | | N/A | |
| RP:5′ CCGCTCGAGTGGCCTACTGTTTCCAGGTC 3′ | | IDT | | N/A | |
| Primer: U2 region  FP:5′ GGGGTACCCCTGGAAACAGTAGGCCAAA 3′ | | IDT | | N/A | |
| RP:5′CCGCTCGAGGTCAGAGCAGCCAAAAGACC 3′ | | IDT | | N/A | |
| Primer: D1 region  FP:5′ GGGGTACCGGGAAGTTTTGCCTCTTCCT 3′ | | IDT | | N/A | |
| RP:5′CCGCTCGAGCTAGCATGCGTGACTGGAGA 3′ | | IDT | | N/A | |
| Primer: D2 region  FP:5′ GGGGTACCCCTGTGTTCCAGCTCTCTCC 3′ | | IDT | | N/A | |
| RP:5′ CCGCTCGAGGGAATCTAGCCAGGGAGGTC 3′. | | IDT | | N/A | |
| ChIP Primers:  BSS1 FP:5′ GAGTGCAATCAGTTTCAGAACAA 3′ | | IDT | | N/A | |
| BSS1 RP:5′ CGTCCAACTTAAAATCCCAAG 3′ | | IDT | | N/A | |
| BSS2 FP:5′ TATTCTGGGTCTCGCCTTG 3′ | | IDT | | N/A | |
| BSS2 RP:5′ GGAATCTAGCCAGGGAGGTC 3′ | | IDT | | N/A | |
| β-actin FP:5′ CACCATTGGCAATGAGCGGTTC 3′ | | IDT | | N/A | |
| β-actin RP:5′ AGGTCTTTGCGGATGTCCACGT 3′ | | IDT | | N/A | |
| p21 FP:5′ GTGGCTCTGATTGGCTTTCTG 3′ | | IDT | | N/A | |
| p21 RP:5′ CTGAAAACAGGCAGCCCAAG 3′ | | IDT | | N/A | |
| Chemicals | |  | |  | |
| Etoposide | | Sigma | | Cat# E1383 | |
| Puromycin | | Sigma | | Cat# P8833 | |
| Dulbecco’s Modified Eagle’s Medium (DMEM) | | Invitrogen | | Cat# 11965118 | |
| Fetal Bovine Serum | | GIBCO | | Cat# 10270106 | |
| Penicillin-Streptomycin | | Invitrogen | | Cat# 15140122 | |
| Lipofctamine-2000 | | Sigma | | Cat# 11668030 | |
| SYBR-Green | | Promega | | Cat# A6001 | |
| Critical Commercial Assays | |  | |  | |
| Dual-Luciferase Reporter Assay System | | Promega | | Cat# E1910 | |
| RNeasy Mini Kit | | QIAGEN | | Cat# 74104 | |
| Q5-Site Directed Mutgenesis Kit | | NEB | | Cat# E0554S | |
| High-capacity cDNA RT Kit | | Applied Biosystems | | Cat# 4368814 | |
| Master Polymer Plus Detection System | | Master Diagnostica | | Cat# MAD-000237QK-10 | |
| Softwares | |  | |  | |
| GraphPad Prism | | v.5.0c | | RRID:SCR_002798 | |
| ImageJ | | v.149 | | RRID:SCR_003070 | |
